# Eliminating Central Line Associated Bloodstream Infections in Pediatric Oncology Patients: A Quality Improvement Effort

**DOI:** 10.1097/pq9.0000000000000660

**Published:** 2023-05-29

**Authors:** Daniel N. Willis, Karen Looper, Rema A. Malone, Barbara Ricken, Ashley Slater, Amanda Fuller, Meagan McCaughey, Angela Niesen, Joan R. Smith, Beverly Brozanski

**Affiliations:** From the * Department of Pediatrics, Division of Hematology/Oncology, Washington University School of Medicine, St. Louis Children’s Hospital, St. Louis, Mo.; † Department of Quality, Safety, and Practice Excellence, St. Louis Children’s Hospital, St. Louis, Mo.; ‡ Department of Hematology/Oncology, St. Louis Children’s Hospital, St. Louis, Mo.; § Department of Pediatrics, Division of Newborn Medicine, Washington University School of Medicine, St. Louis Children’s Hospital, St. Louis, Mo.

## Abstract

**Introduction:**

Central Line-Associated Bloodstream Infections (CLABSI) are the largest contributor to harm across the Children’s Hospital’s Solutions for Patient Safety network. Pediatric hematology/oncology (PHO) patients are at increased risk for CLABSI due to multiple factors. Consequently, traditional CLABSI prevention strategies are insufficient to eliminate CLABSI in this high-risk population.

**Methods:**

Our SMART aim was to reduce the CLABSI rate by 50% from a baseline of 1.89/1000 central line days to less than 0.9/1000 central line days by December 31, 2021. We created a multidisciplinary team being mindful to identify roles and responsibilities upfront. We developed a key driver diagram and designed and implemented interventions to influence our primary outcome.

**Results:**

We implemented interventions and conducted Plan-Do-Study-Act cycles concurrently. We found that performing audits by directly observing tasks rather than auditing documentation resulted in more accurate compliance assessments. As a result, our CLABSI rate improved from 1.89/1000 central line days in 2020 with 11 primary CLABSI to 0.73/1000 central line days in 2021 with four primary CLABSI. Average days between events improved from 30 days in 2020 to 73 days in 2021, and we achieved an unprecedented 542 days CLABSI-free, extending into 2022.

**Conclusions:**

Through a multimodal approach and utilizing characteristics of high-reliability organizations, we significantly reduced primary CLABSI, approaching zero in our PHO population and doubling the average days between events. Future efforts will focus on the sustained engagement of all stakeholders and improving our safety culture.

## INTRODUCTION

Pediatric oncology patients are at high-risk for central line-associated bloodstream infections (CLABSI) due to several factors, including chemotherapy, prolonged need for central venous access, impaired skin and mucosal barriers, and compromised immune system. Comparable to other high-risk populations, the incidence of CLABSI in pediatric oncology patients is around 2.3 infections per 1,000 central line days, significantly higher than in the general pediatric population.^1–3^

CLABSI represents a significant source of morbidity and mortality in oncology patients. Patients who acquire CLABSI require prolonged antibiotic treatment and may require the removal of their central line. Additionally, following CLABSI, patients experience an escalation of care, prolonged hospitalization, and higher healthcare costs.^4^ Nosocomial CLABSI account for up to $55,000 in attributable costs for each episode.^4,5^ In adults, CLABSI is associated with mortality rates as high as 35%.^6^ Although mortality rates are lower in children than adults and less frequently reported, the risk of pediatric mortality is still significant.

During the SARS-CoV-2 pandemic, multiple institutions nationwide have reported increases in CLABSI rates,^7–9^ with some as high as 420% at the beginning of the pandemic.^10^ While this increase temporally is related to the SARS-CoV-2 pandemic, the cause is likely multifactorial. For example, at our institution, CLABSI in children with cancer increased from eight total in the previous two calendar years to nine in the first nine months of 2020.

Multiple interventions aid in CLABSI prevention. Solutions for Patient Safety (SPS), a national collaborative with the mission to eliminate preventable harm from pediatric hospitals, has created and validated a bundle of interventions for both insertion and maintenance of central lines to reduce CLABSI risk.^11,12^ However, because of the unique needs of this population, further efforts are required to eliminate CLABSI.^13^ Bundy et al previously demonstrated that maintenance bundle compliance is not associated with the CLABSI rate in the PHO population.^14^ Preventing CLABSI represents an essential service to our patient population, and this project set out with the global aim to eliminate CLABSI in the pediatric oncology population at our institution.

## METHODS

This improvement project occurred at a large free-standing tertiary care children’s hospital that treats over 200 newly diagnosed oncology patients annually and serves as a destination center with a robust transplant and cellular therapy program. As such, our patient population includes those at the highest risk for CLABSI. The Quality Improvement Oversight Review Committee approved the project, sanctioned by the institutional review board for quality improvement project approval. We followed the SQUIRE 2.0 guidelines in the development of this article.^15^

**Fig. 1.**
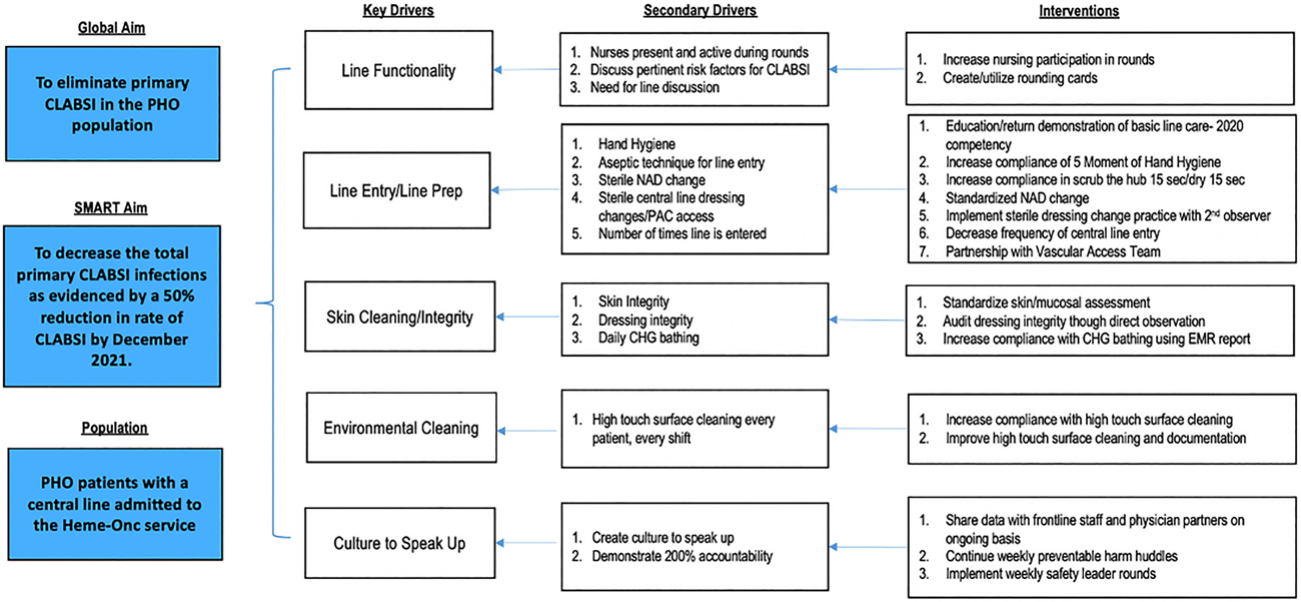
Key Driver Diagram. SIR, standardized infection ratio; NAD, needleless access device; EMR, electronic medical record.

We formed a multidisciplinary team of physicians, nurse practitioners, nursing and physician leadership, frontline staff, and improvement specialists, and we defined roles and responsibilities. We developed a key driver diagram to understand contributing factors and designed interventions that directly and indirectly influenced our primary outcome measure (Fig. 1).

### Outcome Measure

Under the global aim of eliminating all preventable primary CLABSI, our SMART aim was to achieve a 50% reduction in primary CLABSI rate from a baseline of 1.89/1000 central line days to less than 0.9/1000 central line days by December 2021. We defined primary CLABSI using National Healthcare Safety Network standards as any laboratory-confirmed bloodstream infection not seeded from an infection at another body site; therefore, we did not include CLABSI secondary to urinary tract infection, pneumonia, and wounds.^16^ Additionally, we did not include CLABSI caused by intestinal pathogens in patients with risk factors for mucosal barrier injury (classified as MBI-LCBI). As a CLABSI is a relatively rare event, we elected to measure progress by monitoring the CLABSI rate and days between events.

### Process Measures

#### SPS Bundle Compliance

Process measures included SPS Bundle compliance and percent of audits performed by direct observation. We measured compliance as the total number of fully compliant prevention bundle audits over the total number of audits. We recorded any audits with a single noncompliant element as fully noncompliant. Different team members audited the five SPS CLABSI prevention bundle elements on varying days of the week and during different shifts. We set a goal of at least 20 audits per month, which represented approximately 5% of central line days and approximately 20% of encounters for unique patients with central lines. This goal allowed us to trust results were a representative sample of the population. Each month, we displayed the data for frontline staff and faculty to review.

We re-educated auditors on proper technique, and auditors attempted to perform audits by direct observation whenever possible. As such, auditors attended rounds to observe discussion of line, floor leadership audited dressing integrity and line entry technique, and auditors attended dressing changes whenever possible. We measured the total proportion of monthly audits performed by direct observation and designed interventions to concurrently influence multiple areas contributing to CLABSI risk. As these strategies had been insufficient to fully eliminate CLABSI in this population, we designed and implemented additional strategies to achieve our goal (Table 1). Our team ran Plan-Do-Study-Act (PDSA) cycles in tandem.

**Table 1.** Interventions to Reduce CLABSI in Pediatric Oncology Unit

Intervention	Desired Outcome	PDSA Cycle	Start Date
Preventable Harm Huddles	• Increased preoccupation with failure• Improved identification of patients at high-risk for CLABSI	• Modification of scripting for huddle discussion• Defining patient selection criteria	May 2, 2019
Leader Safety Rounds	• Increased SPS bundle compliance• Increased line education for caregivers and patients• Increased accountability for nursing staff	• Rounding with members of physician and nursing leadership• Modifying rounding technique and patient selection	Sep 17, 2020
VA partnership	• Deference to expertise• Improved collaboration with experts• Increased SPS bundle compliance	• VA team performing all SPS audits• VA team partnering with all dressing changes	Oct 1, 2020
HAC rounding cards	• Increased frequency of daily discussion of central lines• Global awareness of central line functionality and concerns	• Implementation with single provider• Implementation on transplant service line• Expansion to full inpatient unit	Sep 14, 2020

VA, Vascular Access.

### Vascular Access Partnership

One identified primary driver was central line entry and central line preparation, including dressing changes. We partnered with our hospital vascular access team to re-educate our staff on central line dressing changes and to perform independent audits of dressing integrity. Although unit nursing staff changed all dressings historically, we began including vascular access team members in each dressing change as expert participants. They served to observe sterile technique, assist in the procedure as necessary, and act as an immediate resource to frontline staff for knowledge and feedback. Immediate feedback is a proven approach to increasing compliance.^17^ Over time, this intervention improved collaboration and deference to expertise, especially in unique or challenging situations.

### Hospital-Acquired Condition (HAC) Rounding Cards

**Fig. 2.**
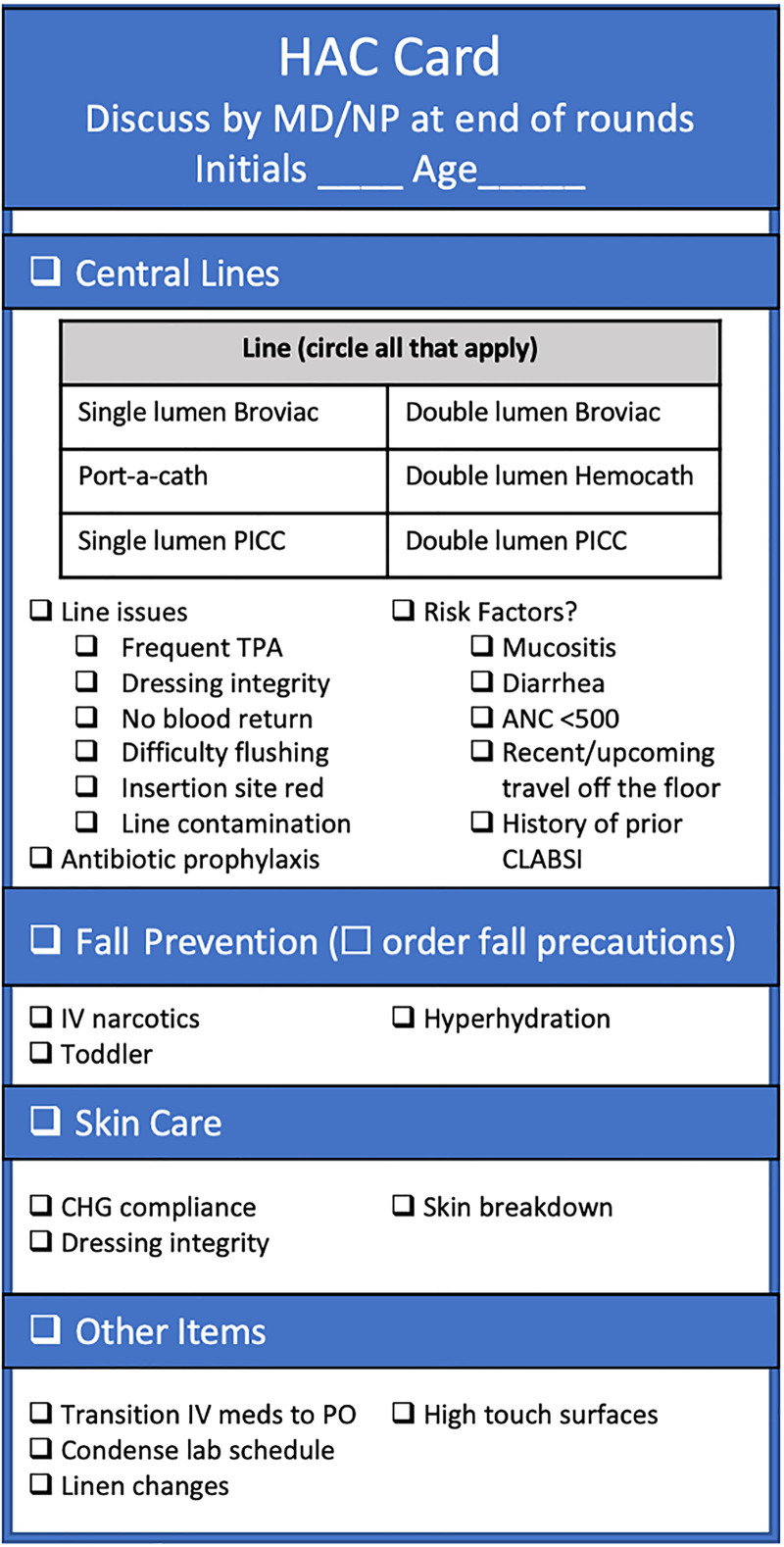
Hospital Acquired Conditions (HAC) Rounding Tool. PICC, peripherally inserted central catheter; TPA, tissue plasminogen activator; IV, intravenous; PO, by mouth.

Daily discussion of line functionality is necessary to identify early issues with line functionality and dressing integrity. To facilitate this discussion, we created a laminated card to be posted in each room (Fig. 2). This card prompts discussion of the line type, need for the line, line functionality and risk factors, and other HAC risks. Before expanding the intervention to all providers, a single provider on the hematopoietic stem cell transplant service trialed the HAC card. PDSA cycles also included prompting HAC rounding card use by CLABSI team members observing rounds, varying the timing of discussion in the overall rounds structure, and audit of its use without verbal prompts. In addition, charge nurses audited the use of HAC cards and line discussion throughout the process.

### Leader Safety Rounds

As part of our effort to add interventions to reduce CLABSI, we implemented weekly rounding by members of divisional quality and safety leadership and nursing leadership. Other centers have demonstrated that multidisciplinary rounds can reduce CLABSI by increasing engagement.^18^ This work began and aligned with the hospital-wide safety goals, and the hospital has adapted and adopted much of this work as standard work. During these rounds, nursing and safety leaders would round on admitted patients with central lines to audit dressing integrity and SPS prevention bundle compliance. In creating a culture of safety, we recognized that a preoccupation with failure was paramount. Therefore, one of the primary goals of this intervention was to recognize and discuss risk factors with parents and nursing. Discussions with parents included proper handling and access of the central line, hand hygiene, and standard components of their child’s care that they could expect during the hospitalization. These discussions established a shared mental model and allowed families to better advocate for their children if care did not meet expectations. PDSA cycles included modifying the content of education, the scope of the audit, the rounding order, and the size of the rounding team. Ultimately, we established a rounding team of three members who would enter the room with the bedside nurse to assess the central lines and tubing and provide education on dressing integrity, line entry, chlorhexidine gluconate (CHG) bathing, and hand hygiene. We chose patients based on nursing assignments to maximize the number of nurses participating. As a result, we could include roughly half of the nurses on the unit while completing 6-7 audits in an hour. Over a month, most of our core nursing staff had participated at least once. Each time, we monitored bundle compliance and the number of patients on which we rounded.

### Preventable Harm Huddles

With a preoccupation with failure mindset, our team identified patients at the highest risk for CLABSI to discuss in a huddle format. This intervention was originally implemented in 2019, before project initiation. While we had not yet seen improvement, we felt it was valuable to identify our highest-risk patients, and it aligned well with our key driver of “culture to speak up.” For this reason, we recommitted to continuing Preventable Harm Huddles as part of this project. Distinct from Leader Safety Rounds, during a Preventable Harm Huddle, we would discuss all risk factors affecting a single patient for awareness and to develop patient-specific mitigation strategies. Frontline staff identified patients based on the history of CLABSI, degree of immunosuppression, type of central line, skin and oral mucosa integrity, and nursing needs. Weekly huddles included unit safety specialists, nursing leadership, physician leadership, infection prevention team members, vascular access team members, family members, and frontline nursing and providers. PDSA cycles included changes to the scripting and structure of the huddle. The standard structure of huddles included a discussion of central line bundle compliance adherence, environmental cleanliness, central line utilization, risk and symptoms of mucosal barrier injury and degree of immunosuppression. We completed huddles within 30 minutes and incorporated any preventative action items that resulted from the huddle into the patient’s care.

### Analysis

To calculate baseline average rates for process and outcome measures, we used 12 data points and plotted these rates on run charts. A U chart captured monthly CLABSI rates per 1000 central line days, and we plotted the days between events on a T chart. We demonstrated compliance with the SPS CLABSI maintenance bundle on a P chart. Using run chart rules outlined by Perla et al, eight points above or below the mean constituted a run and triggered the calculation of a new baseline. To make inferences regarding causality, we annotated charts as interventions occurred.^19^

## RESULTS

**Fig. 3.**
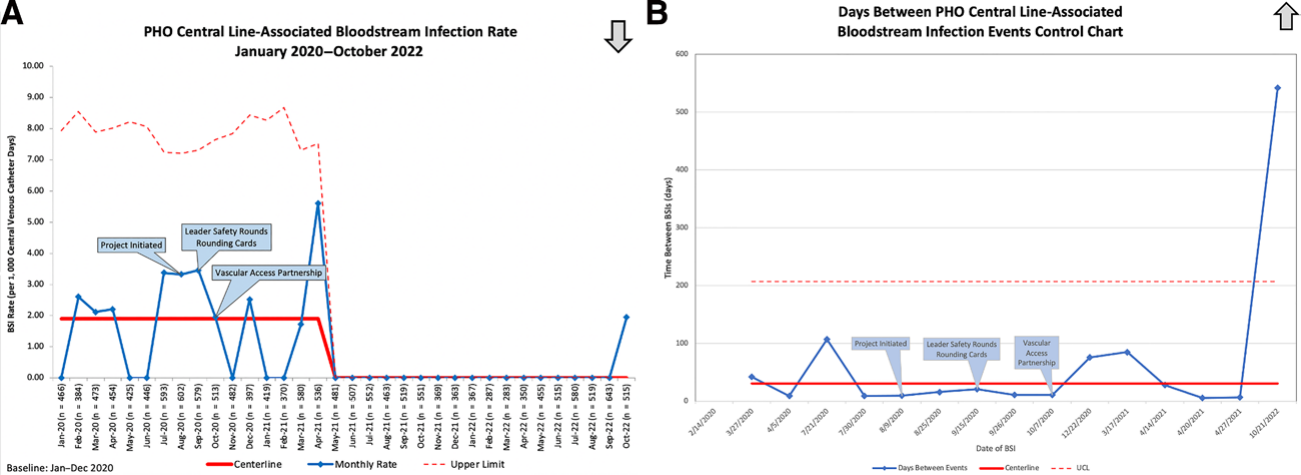
A, Monthly CLABSI Rate per 1000 Central Venous Catheter Days. BSI, bloodstream infection. *Preventable Harm Huddles implemented before project initiation. B, T Chart: days between PHO CLABSI events. BSI, bloodstream infection; UCL, upper control limit. *Preventable Harm Huddles implemented before project initiation.

In 2020, we recorded 11 primary CLABSI with 5814 central line days, for a CLABSI rate of 1.89 infections per 1000 central line days. In 2021, we recorded four primary CLABSI with 5710 central line days, resulting in a CLABSI rate of 0.7 per 1000 central line. In 2022, we recorded over 4000 central line days before recording a primary CLABSI. Figure 3A shows our control chart with the monthly CLABSI rate. Our baseline CLABSI rate in 2020 was 1.89 per 1000 central line days. After eight months free of CLABSI, the centerline shifted to 0, where it has remained. During this period, there were 5 MBI CLABSI in 2020, four in 2021, and 8 in 2022.

Figure 3B documents the days between CLABSI events in a T chart. The centerline is 38 based on the initial 12 data points beginning in 2020. The average days between events in 2020 was 30, improving to 73 average days in 2021. The days between events spanned an institutional record of 542 in 2021 and 2022.

**Fig. 4.**
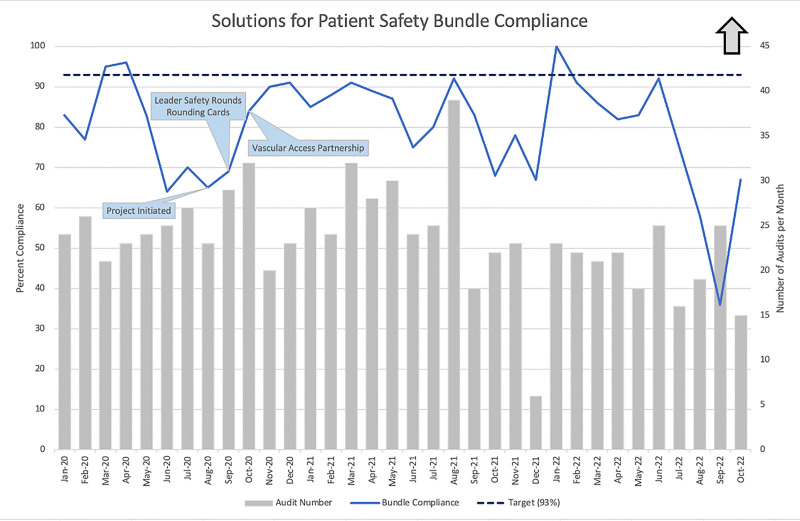
Process Measure – SPS Bundle Compliance. *Preventable Harm Huddles implemented before project initiation.

Figure 4 shows our process measure of SPS CLABSI prevention bundle compliance with monthly audits and percent compliance and annotated interventions. Compliance had wide variation by month, influenced in part by sample size. We presented monthly data to frontline staff and providers for awareness. Over 6 months of implementation, Preventable Harm Huddles occurred weekly, 80% of the time. When huddles could not occur, reasons included holidays and staffing shortages requiring nursing leadership to aid in patient care. Preventable Harm Huddles and Leader Safety Rounds both continue to date.

We did not see an immediate decline in CLABSI after our interventions; however, with continued reinforcement, the CLABSI rate began to fall. Days between events showed a more immediate improvement, with an average of 63 days between events for the 3 CLABSI after all interventions compared with 13 days for the 6 events before and during interventions. Additionally, our process measure saw a fall in monthly audits, with a rise in the compliant percentage.

## DISCUSSION

This quality improvement initiative demonstrates successful interventions in CLABSI prevention in a large tertiary free-standing children’s hospital academic PHO program. Our program cares for a diverse patient population with significant risk factors and immunosuppression. In the last 11 years, we have never had a length of time CLABSI-free that rivals the current stretch. Of course, not all CLABSI are preventable, but the goal of “CLABSI free” with rates approaching zero is attainable and sustainable with our described improvement efforts. Our hospital patient safety team investigates contributing factors with frontline staff and infection prevention leaders for each bloodstream infection. We approached the issue in many directions by forming a multidisciplinary team focused on preventing CLABSI. We designed additional interventions of direct observations, Preventable Harm Huddles, and Leader Safety Rounds, ultimately eliminating primary CLABSI. We speculate that this decrease in CLABSI led to a decrease in antibiotic use, hospital length of stay, and, ultimately, a decrease in healthcare costs and improved quality of care.

The increase in CLABSI in 2020 was likely multifactorial and due to extrinsic global factors. We made no institution-specific structural or programmatic changes during this period that contributed to the rise or subsequent improvement in CLABSI. There was also no variation in the inpatient patient composition or average census. However, the Sars-CoV-2 pandemic gave rise to many difficulties. Global supply disruptions resulted in the intermittent need to substitute products. For example, central line dressing kits were often incomplete and did not always include chlorhexidine scrub pads. As a result, the nurse had to open the kit and assess what supplies were needed, remove and replace sterile gloves to obtain those items, or request supplies. This change from the standard process, during the pandemic, with a high rate of nursing turnover and an increase in contingency staff and onboarding of nurses with less experience, contributed to additional practice variation. Unit rounding also changed due to virtual capacity and social distancing. These changes disrupted operational standards and communication, essential characteristics of high-reliability organizations (HROs). Similarly, our improvement was multifactorial, with no clear improvement immediately after any single intervention. However, we began to see improvement over time and with subjective changes in culture and dressing integrity.

Many of our efforts were related to characteristics of HROs, with a preoccupation with failure being central to the work. Essential characteristics of HROs include deference to expertise, preoccupation with failure, reluctance to simplify, commitment to resilience, and sensitivity to operational standards.^20^ By implementing Leader Safety Rounds and Preventable Harm Huddles, we partnered with caregivers, physicians, frontline staff, and patients/families to convey a shared model of responsibility and vigilance and to align understanding of prevention strategies. By partnering with the vascular access team, we leaned into a model of deference to expertise and sensitivity to operational standards. In addition, we demonstrated a reluctance to simplify and a revived focus on the processes by utilizing a direct observation approach to compliance audits. Perhaps most important to this effort was a commitment to resilience. Our team takes great pride and personal responsibility in caring for the patients, and each CLABSI is taxing and often taken personally by the staff. Therefore, we had to make a sustained effort to maintain leadership presence and refocus the conversation on the processes to maintain a culture of safety. Anecdotally, there was initial hesitation to participate by frontline staff. However, there was a noticeable shift over time, with increased receptiveness as the culture was reinforced.

One difference in observed versus expected outcomes includes prevention bundle compliance audit results. Throughout this project, we saw relatively static bundle compliance results. Similarly, bundle compliance did not directly correlate with our increase in primary CLABSI during the pandemic. We believe this is due to the audit accuracy of the work performed. By directly visualizing the prevention elements, we could give feedback in the moment for deficiencies. We documented these as noncompliant for audit purposes. It is possible that in the past, documentation was variable for noncompliant elements that auditors corrected in the moment. Thus, bundle compliance failed to show a decrease when all elements were not correctly completed. Similarly, bundle compliance remained static and even fell when subjectively, we were making improvements. Ultimately, direct observation of bundle compliance and discussion of line functionality refocused our team on the importance of these bundle elements and led to sustained improvement in practice.

This quality improvement initiative has several factors that may limit generalizability; however, any hospital inpatient unit can apply many of the interventions. The patient population comprises pediatric hematology, oncology, and cellular therapy patients in a single unit. While the interventions are not unique to this population, some may require modification based on population characteristics. For pediatric hematology/oncology patients, our patients uniformly receive pneumocystis prophylaxis, and select populations (stem cell transplant, acute myeloid leukemia and all relapsed leukemia patients) receive bacterial prophylaxis during periods of neutropenia. No changes to this prophylactic regimen or inclusion criteria occurred during the project. However, prophylactic antibiotics may influence the spectrum of infections seen in patients with central lines. We believe implementing a single unit added to this project’s success as we continuously reinforced efforts with the same core group of providers, nurses, patients, and caregivers. Over time, the majority of patients and staff participated.

Similarly, implementation on a single unit allowed for direct engagement from unit leadership. By customizing interventions, other units and hospitals can generate similar enthusiasm and interest to add these elements to standard safety work. As characteristics vary between hospital units, we believe that having buy-in and unit-based nursing and physician champions would be necessary for hospital-wide implementation so that each unit could tailor interventions to their needs. Our hospital has a dedicated vascular access team with the bandwidth to regularly assist with audits and central line dressing changes. To scale this project hospital-wide, unit leadership would need to assess required resources and validate the processes unique to each unit. This effort required personnel resources which proved challenging given the nationwide staffing crises. However, as we incorporated interventions into standard work, this burden lessened. If repeated, a balancing measure to assess task load would be helpful.

In summary, by forming a multidisciplinary team, modeling characteristics of HROs, and maintaining constant vigilance, we have demonstrated a sustained decline in CLABSI in a high-risk population and an unprecedented stretch without a primary CLABSI. We speculate that this success has widespread implications for quality of care, decreased morbidity, and decreased mortality. Future efforts will focus on adapting interventions to encompass CLABSI prevention throughout our institution.

## ACKNOWLEDGMENT

We wish to thank the St. Louis Children’s Hospital vascular access team, the Infection Prevention Subcommittee, and the St. Louis Children’s Hospital Quality Improvement Bootcamp Faculty, without whom we would have been unable to complete this work.

## DISCLOSURE

The authors have no financial interest to declare in relation to the content of this article.
